# *Prevotella* Induces the Production of Th17 Cells in the Colon of Mice

**DOI:** 10.1155/2020/9607328

**Published:** 2020-11-01

**Authors:** Yuan Huang, Jinhua Tang, Zheng Cai, Keqiang Zhou, Liang Chang, Yinlan Bai, Yueyun Ma

**Affiliations:** ^1^Department of Clinical Laboratory, The First Affiliated Hospital, Air Force Medical University, Xi'an, Shaanxi Province 710032, China; ^2^The Second Clinical Medical College, Shanxi Medical University, Taiyuan, Shanxi Province 030001, China; ^3^Department of Microbiology and Pathogen Biology, Basic Medical School, Air Force Medical University, Xi'an, Shaanxi Province 710032, China; ^4^Department of Clinical Laboratory, Air Force Medical Centre, Beijing 100142, China

## Abstract

Th17-mediated mucosal inflammation is related to increased *Prevotella* bacterial abundance. The actual involvement of *Prevotella* in the development and accumulation of intestinal Th17 cells at a steady state, however, remains undefined. Herein, we investigated the role of *Prevotella* in inducing intestinal Th17 cells in mice. Mice were treated with a combination of broad-spectrum antibiotics (including ampicillin, neomycin sulfate, vancomycin hydrochloride, and metronidazole) in their drinking water for 4 weeks and then gavaged with *Prevotella* for 4 weeks. After inoculation, 16S rDNA sequencing was used to verify the colonization of *Prevotella* in the colon of mice. The IL-17A as well as IL-17A-expressing T cells was localized and quantified by an immunofluorescence assay (IFA) of colon sections. Th17 cells in the mesenteric lymph nodes of mice were counted by flow cytometry. Systemic immune response to *Prevotella* colonization was evaluated based on the serum levels of IL-6, TNF-*α*, IL-1*β*, IL-17A, IL-10, IL-4, IFN-*γ*, and IL-2. Th17-polarizing cytokines (IL-6, TNF-*α*, IL-1*β*, and IL-2) induced by *Prevotella* were evaluated by stimulation of bone marrow-derived dendritic cells (BMDCs). Results revealed that after inoculation, *Prevotella* successfully colonized the intestine of mice and induced the production and accumulation of colonic Th17 cells in the colon. Moreover, *Prevotella* elevated some of the Th17-related cytokines in the serum of mice. And Th17-polarizing cytokines (IL-6 and IL-1*β*) produced by BMDCs were mediated mainly through the interaction between *Prevotella* and Toll-like receptor 2 (TLR2). In conclusion, our data suggest that *Prevotella* induces the production of Th17 cells in the colon of mice, thus highlighting the potential role of *Prevotella* in training the intestinal immune system.

## 1. Introduction

The central role of the microbiota in human disease and health is gaining more attention since it can shape host immune development and modulate host immune responses [1]. Specific intestinal microbes have been suggested to regulate the homeostasis of intestinal effector T cells. For example, certain bacterial species from *Bacteroides* and *Clostridium* have been identified to induce regulatory T cells in the murine colon [2–5]. Another study showed that a subgroup of microbes in the intestine, such as segmented filamentous bacteria (SFB), *Citrobacter rodentium*, and *Escherichia coli* O157, can induce the production of Th17 cells in the intestine [6].

Th17 cells are prominent among T cells present in the intestines of both mice [7, 8] and humans [9]. The Th17 cytokines, IL-17A, IL-17F, and IL-22, induce the intestinal epithelium to produce tight junction proteins and antimicrobial peptides, supporting the integrity of the gut barrier [10]. In addition, IL-17A and IL-17F recruit neutrophils by releasing granulocyte colony-stimulating factor, thereby helping host fight against extracellular bacterial and fungal infections [11]. As a result, humans with IL-17 signalling defects are more susceptible to mucosal infections with *Staphylococcus aureus* and *Candida albicans* [11, 12]. However, excessive Th17 responses have been associated with a variety of autoimmune and inflammatory disorders [12, 13]. Recently, there is epidemiological evidence for the relationship between *Prevotella* and increased Th17-mediated immune responses in many inflammatory diseases [14–16].

*Prevotella* strains are gram-negative anaerobes that are members of the gut, oral, and vaginal microbiota [17]. In human gut microbial communities, as one of the three gut enterotypes [18, 19], *Prevotella* abundance is associated with chronic inflammatory conditions [20], as well as plant-rich diets [21]. Of note, emerging researches in humans have associated the enrichment of *Prevotella* in the mucous membrane with Th17-related inflammatory diseases, including bacterial vaginosis [22, 23], periodontitis [15], and rheumatoid arthritis [16, 20, 24]. This is consistent with the marked ability of *Prevotella* to induce Th17 *in vitro*. However, it is unclear whether *Prevotella* has a similar effect on Th17 cells in the absence of inflammatory diseases *in vivo*. In other words, there is still no direct evidence that *Prevotella* induces colonic Th17 cells. Therefore, we set out to assess the role of *Prevotella* in the induction of Th17 cells in the colon of mice.

## 2. Materials and Methods

### 2.1. Experimental Animals and Microbial Strains

L-17A^˗/˗^ (KO) mice in the C57BL/6 background were purchased from the Shanghai Model Organisms Center (Shanghai, China), and age-matched C57BL/6 (WT) mice were from the Department of Lab Animal Science of Air Force Medical University. All mice were maintained in groups of 5 animals per cage under specific pathogen-free conditions at the Department of Lab Animal Science of Air Force Medical University. Unless otherwise stated, 6–8-week-old female mice were used. The protocols for animal experiments were approved by the Laboratory Animal Welfare and Ethics Committee of Air Force Medical University (no. KY20173518-1), and all experiments were performed following the relevant guidelines. For the stimulation of BMDCs, *Prevotella copri* (DSMZ 18205) and *Prevotella melaninogenica* (ATCC®25845™) were cultured on Columbia blood plates (Oxoid) at 37°C under anaerobic conditions for 3 days prior to adjusting the concentration to an OD600 nm of ~0.5 [15].

### 2.2. Antibiotic Treatment and *Prevotella* Inoculation

Ampicillin (1 g/L, Amresco), neomycin sulfate (1 g/L, Amresco), vancomycin hydrochloride (0.5 g/L, Vancocin), and metronidazole (1 g/L, Alfa Aesar) were added into the drinking water (ABX) of the mice for 4 weeks [25]. Microbial depletion was confirmed by examining the presence of living microorganisms in aerobic or anaerobic culture. Water containing antibiotics was changed twice a week, and treatment was stopped 2 days prior to gavage of *Prevotella*. For preparation of the bacterial inoculum, *Prevotella* was grown on fluid thioglycolate medium (Oxoid) at 37°C under anaerobic conditions for 3 days before use. After centrifugation, the bacteria were suspended in the fluid medium. Mice were gavaged with 200 *μ*L of inoculum (dose 1 × 10^8^) and received the doses every other day for 4 weeks as previously described [15, 26]. One hour prior to the bacterial gavage, mice were injected intraperitoneally with 3 mg of cimetidine HCl (Sigma-Aldrich) in 100 *μ*L PBS to inhibit stomach acid secretion to improve the colonization [26].

### 2.3. 16S rDNA Sequencing Analysis of Mouse Faeces

Fresh faeces were collected aseptically from mice prior to euthanization and were stored at ˗80°C before analysis. Genomic DNA extraction from faeces was performed using the QIAamp PowerFecal DNA Kit (Qiagen). The 16S ribosomal DNA hypervariable regions V3+V4 were PCR-amplified using primers 338F ACTCCTACGGGAGGCAGCAG and 806R GGACTACHVGGGTWTCTAAT. All PCR reactions were carried out on an ABI GeneAmp® 9700 (Thermo Fisher) with Trans Start Fastpfu DNA Polymerase (TransGen). The PCR products were purified with the AxyPrep DNA Gel Extraction Kit (Axygen). Sequencing libraries were generated using the TruSeq DNA Sample Prep Kit (Illumina) and sequenced on an Illumina Miseq PE300 platform (Illumina) following the manufacturer's recommendations. The Ribosomal Database Project (RDP) classifier (version 2.11) Bayes algorithm was used to annotate the taxonomic information of operational taxonomic units (OTUs) with ≥97% similarity. And the relative abundance of each OTU was calculated at each classification level (kingdom, phylum, class, order, family, and genus). The composition of the gut microbiota was further analyzed as previously described [27].

### 2.4. Quantification of Cytokines in the Serum

Blood was collected from the eyes of the mice and was allowed to clot for at least 30 min before centrifugation for 10 min at 1000 × *g*. Then, the serum was removed and assayed on a multiplex LUMINEX xMAP MAGPIX instrument (Millipore Corporation). Antibody beads, controls, wash buffer, serum matrix, and standards were prepared for the MILLIPLEX® MAP Kit Mouse Th17 Magnetic Bead Panel (Millipore Corporation) following the manufacturer's instructions [28]. Concentrations of eight cytokines (IL-6, TNF-*α*, IL-1*β*, IL-17A, IL-10, IL-4, IFN-*γ*, and IL-2) were detected using the Mouse Th17 Magnetic Bead Panel according to its instructions. MAGPIX and xPONENT software were used to read the results.

### 2.5. Bacterial DNA and Real-Time PCR

Faecal DNA was extracted according to instructions (QIAamp Fast DNA Stool Kit, Qiagen). Phylum-specific primers were used to detect 16S rDNA by real-time PCR in triplicate [25] (Table S1) with a ChamQ™ SYBR® Green PCR Master Mix (Vazyme) performed on a Bio-Rad CFX96 System. The universal 16S rDNA gene was used to normalize the values, and the data were calculated using the 2^-*ΔΔ*CT^ method as previously described [29]. The expression of multiple changes in the experimental samples (ABX-treated group or *Prevotella*-gavaged group) was compared with that in the control samples (non-ABX-treated group).

### 2.6. Cellular RNA Isolation and Real-Time RT-PCR

Total RNA from mouse colons was isolated with the TRIzol reagent (Invitrogen) after homogenization of the tissue. RNA was reverse transcribed using a HiScript® III RT SuperMix for qPCR (+gDNA wiper) Kit (Vazyme). Real-time PCR analyses were performed in triplicate on the Bio-Rad CFX96 System with a ChamQ™ SYBR® Green PCR Master Mix (Vazyme) using gene-targeted primers (Table S1). The *gapdh* housekeeping gene values were used to normalize the values, and the data were calculated using the 2^-*ΔΔ*CT^ method as previously described [29]. The expression of multiple changes in the experimental samples (ABX-treated group) was compared with that in the control samples (non-ABX-treated group).

### 2.7. Bone Marrow-Derived Dendritic Cell Generation and Stimulation

Bone marrow-derived dendritic cells (BMDCs) were obtained as previously described [30]. Briefly, bone marrow cells were isolated from 6- to 8-week-old C57BL/6 mice and cultured for 9 days with 20 ng/mL recombinant murine GM-CSF (PeproTech). Cells were purified by positive selection [26] using anti-CD11c microbeads (Miltenyi Biotec). The dendritic cell (DC) phenotype was controlled by flow cytometry. The cytokine response to *Prevotella* was assessed in 1 × 10^5^ BMDCs incubated with LPS (10 ng/mL, Sigma) as a positive control, PBS as a negative control, or heat-killed (30 min at 60°C) *Prevotella* (1 × 10^7^ cfu/well) for 24 h [15]. To compare the efficacy of Toll-like receptors (TLRs), the BMDC medium was supplemented with inhibitors of TLR2 [31] (C29, MCE, 50 *μ*M) or TLR4 [32] (TAK-242, MCE, 100 nM). For all experiments, TLR inhibitors were added to the cells just prior to stimulation.

### 2.8. Cell Isolation and Flow Cytometry

Mice were sacrificed, and mesenteric lymph nodes (MLNs) were harvested aseptically. Then, MLNs were homogenized using a syringe and filtered on 40 *μ*m cell strainers to make single-cell suspensions. For intracellular cytokine staining, cells were incubated for 4 h with ionomycin and phorbol myristate acetate (PMA) (1 *μ*g/mL and 50 ng/mL, respectively, Sigma-Aldrich) at 37°C under 5% CO_2_ [26]. Antibody staining was performed at 4°C for 30 min. Antibodies raised against the following mouse antigens were used: IL-17A (clone REA660, Miltenyi Biotec), CD4 (clone GK1.5, Miltenyi Biotec), MHC II (clone M5/114.15.2, Miltenyi Biotec), and CD11c (clone N418, Miltenyi Biotec). Intracellular staining was done with an Inside Stain Kit (Miltenyi Biotec) according to the manufacturer's instructions. Flow cytometry was performed on a BD FACSCanto™ II Flow Cytometer, with data subsequently analyzed with FlowJo software (Tree Star).

### 2.9. ELISA Analysis of Cytokines

Supernatant from stimulated BMDC cultures was collected and analyzed using Boster ELISA kits for cytokine concentrations, including IL-1*β*, IL-6, IL-12p70, and TNF-*α*. The detection procedures were carried out according to the instructions. Data were expressed as the mean cytokine response minus the background (pg/mL) for each treatment from triplicate wells.

### 2.10. Immunofluorescence Assay

Colon samples were embedded in O.C.T. (Sakura Finetek), cut in 4 *μ*m sections, and adhered to microscope slides (Thermo Fisher Scientific). All slices were blocked with 100 *μ*L of blocking solution for 30 min at room temperature in a humidified chamber. Then, the primary and secondary antibodies were incubated in a humidified chamber each at room temperature for 60 min. Phosphate-buffered saline (pH 7.4) was used to wash the slices following the primary and secondary antibody incubations three times for 5 min. And sections were counterstained with DAPI. The primary antibodies used were monoclonal rabbit anti-IL17A (Abcam) and monoclonal rat anti-CD3 (Abcam), respectively, and both were diluted to 1 : 200 in antibody dilution buffer (Solarbio). The primary antibodies were fluorescently labelled separately with Alexa Fluor 488 goat anti-rabbit IgG (Abcam) and Alexa Fluor 647 goat anti-rat IgG (Abcam) secondary antibodies, that were diluted to 1 : 1000 in antibody dilution buffer (Solarbio).

### 2.11. Quantification and Statistical Analysis

Statistical analyses were performed using GraphPad Prism 8 software. Data obeying a normal distribution are represented as mean ± SD and analyzed using the unpaired *t*-test (when two samples were compared) or ANOVA (when more than two samples were compared); data obeying a nonnormal distribution are represented as median with interquartile range and analyzed using the Mann-Whitney (when two samples were compared) or Kruskal-Wallis (when more than two samples were compared) tests. Differences were considered significant when *p* < 0.05. Mice were randomly assigned into experimental groups with 5 mice per group.

## 3. Results

### 3.1. Depletion of Intestinal Flora Reduced *il-17a* Gene Transcription and IL-17A-Positive T Cells in the Colon

**Figure 1:**
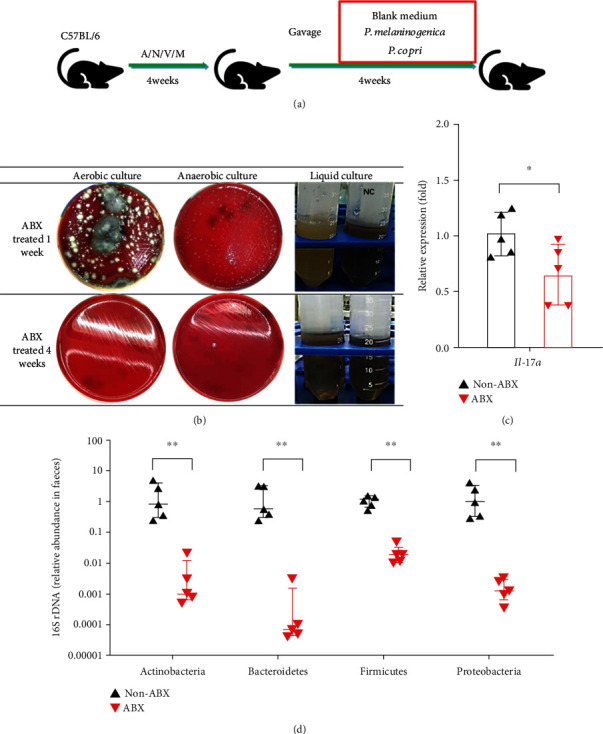
ABX treatment reduced both gut microbial abundance and mRNA expression of *il-17a* in the colon. (a) Experimental design. Six- to eight-week-old C57BL/6 female mice were treated for 4 weeks with a cocktail of broad-spectrum antibiotics (ampicillin, neomycin sulfate, vancomycin, and metronidazole) in drinking water (ABX) or were given water without antibiotics (non-ABX). Then, the ABX-treated mice were divided into 3 groups: the WT+BM group was gavaged with blank medium for 4 weeks, the WT+PM group was gavaged with *P. melaninogenica* for 4 weeks, and the WT+PC group was gavaged with *P. copri* for 4 weeks. (b) Evaluation of the presence of microbial flora in the faeces of ABX-treated mice by culture in either aerobic or anaerobic conditions. (c) Colonic *il-17a* mRNA expression in the ABX vs. non-ABX groups. The graph represents mean ± SD, and data were analyzed using the unpaired Student's *t*-test. (d) The faecal microbiota was detected by qPCR using phylum-specific primers. Relative Ct value compared to universal 16S rRNA gene Ct value (*∆*Ct) and the mean of *∆*Ct values in the non-ABX group (*∆∆*Ct). The graph shows the median with interquartile range, and data were analyzed using the Mann-Whitney test. Statistical significance is displayed as ^∗^*p* < 0.05 and ^∗∗^*p* < 0.01.

As is known to all that germ-free mice [33], rather than antibiotic-treated mice [34], exhibit abnormal immune functions, so we used the ABX-treated mice for the investigation of whether *Prevotella* could induce colonic Th17 development *in vivo*. Mice were treated with ABX for 4 weeks to deplete commensal organisms in the gut and then gavaged with *P. melaninogenica* (WT+PM), *P. copri* (WT+PC), or blank medium (WT+BM) every other day for 4 weeks (Figure 1(a)).

The depletion effect on intestinal flora was verified by aerobic, anaerobic, and fluid culture of mouse faeces (Figure 1(b)). As the culture results showed, after being treated with ABX for 4 weeks, mouse faeces contained virtually no culturable bacteria (Figure 1(b)). Furthermore, faecal microbiota composition at the phylum level detected by qPCR using phylum-specific primers indicated that the relative abundance of four major phyla including Actinobacteria, Bacteroidetes, Firmicutes, and Proteobacteria all decreased significantly after ABX treatment (Figure 1(d)).

**Figure 2:**
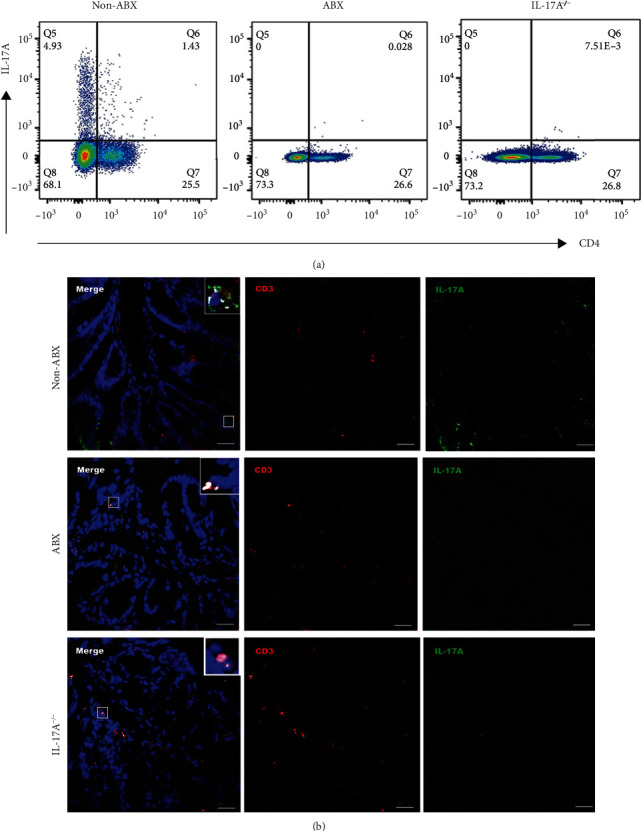
ABX treatment reduces Th17 in the colon of mice. (a) Expression of IL-17A in CD4^+^ cells from the mesenteric lymph nodes (MLNs) of the WT mice treated with or without ABX, as well as the IL-17A^−/−^ mice. (b) Immunofluorescence assay (IFA) of sections of the colon in the WT mice treated with or without ABX, as well as the IL-17A^−/−^ mice. The colon slices were stained with monoclonal antibodies of IL-17A (green) and CD3 (red). And DAPI (blue) stained the nucleus. Scale bar: 20 *μ*m.

Moreover, *il-17a* mRNA expression in the mouse colon reduced dramatically after the depletion of intestinal flora (Figure 1(c)). And the results of flow cytometry revealed that the proportion of Th17 cells from MLNs decreased dramatically after ABX treatment (Figure 2(a)). Consistently, the immunofluorescence assay (IFA) showed that colonic T cells coexpressing IL-17A and CD3 decreased after ABX treatment (Figure 2(b)). Of interest, the lack of IL-17A-expressing T cells in ABX-treated mice was similar to that in IL-17A^−/−^ mice (Figure 2(b)). These results indicate that the intestinal microbiota is indispensable for the development of colonic Th17 cells.

### 3.2. *Prevotella* Induced IL-17A Production and Th17 Cell Accumulation in the Colon of Mice

**Figure 3:**
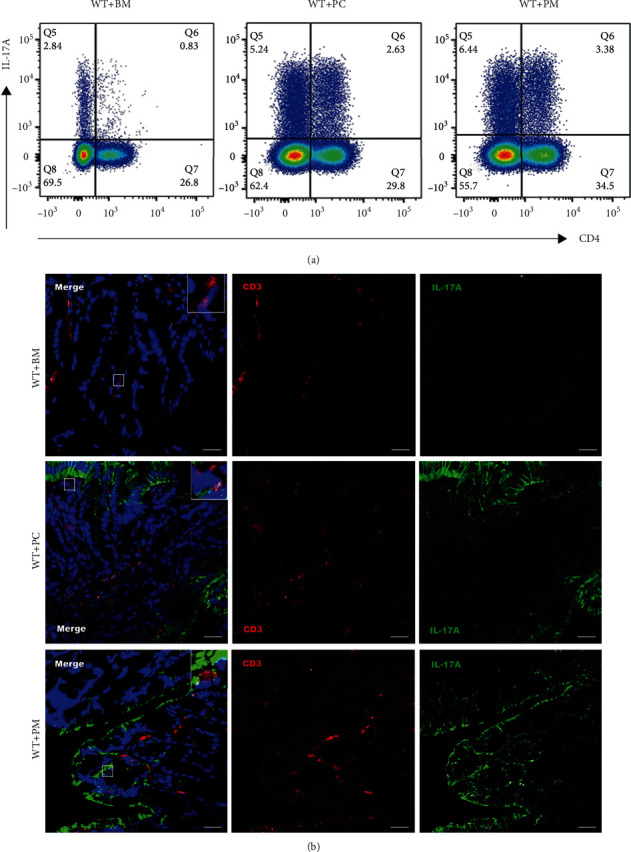
*Prevotella* inoculation augmented Th17 development in the colon of mice. (a) Expression of IL-17A in CD4^+^ cells from the MLNs of the ABX-treated WT mice gavaged with blank medium, *P. copri*, and *P. melaninogenica*, respectively. (b) IFA of colon segments of the ABX-treated WT mice gavaged with blank medium, *P. copri*, and *P. melaninogenica*, respectively. The colon slices were stained with monoclonal antibodies of IL-17A (green) and CD3 (red). And DAPI (blue) stained the nucleus. Scale bar: 20 *μ*m.

After verifying the depletion of the intestinal microbiota, we gavaged ABX-treated mice with *P. melaninogenica* (WT+PM), *P. copri* (WT+PC), or blank medium (WT+BM), respectively, for 4 weeks (Figure 1(a)). Compared with WT+BM, both WT+PM and WT+PC groups showed significant elevation of Th17 cells in the MLNs (Figure 3(a)). Similarly, proportions of colonic cells coexpressing CD3 and IL-17A of the two *Prevotella*-gavaged groups (WT+PM and WT+PC) were higher than that of the blank medium-gavaged control group (WT+BM) (Figure 3(b)). Of note, the IFA results showed clearly that the majority of IL-17A is distributed in the intestinal epithelial cells, indicating its major effects on them (Figure 3(b)).

**Figure 4:**
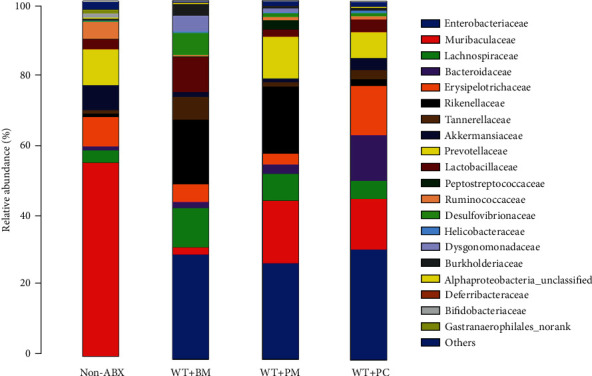
*Prevotella* successfully colonized the intestine of mice after its inoculation. Gut microbial community compositions in mice from different groups were analyzed at the family level by 16S rDNA sequencing.

To determine whether the induction of Th17 cells authentically depends on the inoculation of *Prevotella* in the current study, we checked the microbial community compositions of faeces from the WT+PM, WT+PC, WT+BM, and non-ABX mice by using 16S rDNA sequencing. The results revealed relatively higher proportions of *Prevotellaceae* in the microbiota of WT+PM and WT+PC mice compared to WT+BM mice (Figure 4). Besides, relative abundances of four major phyla (Actinobacteria, Bacteroidetes, Firmicutes, and Proteobacteria) in the faecal microbiota from WT+PM, WT+PC, and WT+BM recovered asynchronously from ABX treatment (Figure S1).

The 16S rDNA sequencing analysis indicated that both ABX treatment and *Prevotella* inoculation changed the compositions of the intestinal microbiota of mice and that *Prevotella* colonized successfully after its inoculation. Combined with the accumulation of colonic Th17 cells after *Prevotella* inoculation, the induced development and accumulation of Th17 cells definitely resulted from the colonization of *Prevotella* in the colon of mice.

### 3.3. *Prevotella* Partly Elevated Th17-Related Cytokines in the Serum of Mice

**Figure 5:**
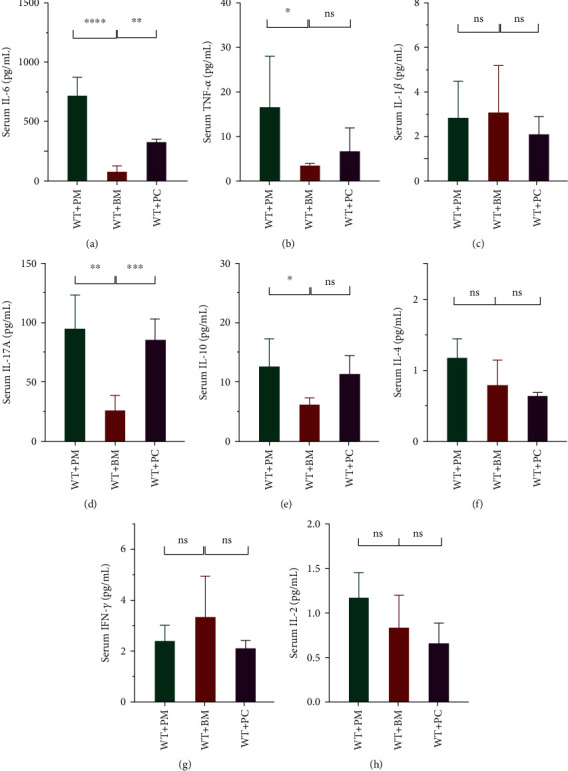
*Prevotella* elevated Th17-related cytokine levels in the serum of mice. (a–h) Serum cytokines (IL-6, TNF-*α*, IL-1*β*, IL-17A, IL-10, IL-4, IFN-*γ*, and IL-2) were quantified in the three groups of mice. Data are presented as the mean ± SD. The *p* values were calculated using one-way ANOVA. Statistical significance is displayed as ^∗^*p* < 0.05, ^∗∗^*p* < 0.01, ^∗∗∗^*p* < 0.001, and ^∗∗∗∗^*p* < 0.001.

Since Th17 cells exhibit a proinflammatory effect, we seek to determine whether intestinal Th17 expansion in *Prevotella*-colonized mice was accompanied by inflammatory response. Results of serum cytokine detection showed that *P. melaninogenica* colonization augmented the serum concentration of IL-6 and TNF-*α* while *P. copri* colonization only elevated IL-6 in serum (Figures 5(a) and 5(b)), whereas the concentration of IL-1*β* showed no significant difference between the three groups (Figure 5(c)). It's no surprise that compared with the WT+BM group, *P. melaninogenica* and *P. copri* colonization elevated the serum IL-17A significantly (Figure 5(d)). Other serum cytokines including IL-4, IFN-*γ*, and IL-2 remained stable after the colonization of *Prevotella*, except for the increase in IL-10 related to *P. melaninogenica* colonization (Figures 5(e)–5(h)). Analysis of serum cytokines revealed that *Prevotella* colonization mainly promoted Th17-related cytokines (i.e., IL-17A and IL-6) and had no effect on the Th1- (i.e., IFN-*γ* and IL-2) and Th2- (i.e., IL-4) related cytokines. Meanwhile, the effect of *P. melaninogenica* colonization on serum cytokines was more extensive than that of *P. copri*.

### 3.4. *Prevotella* Activates TLR2-Mediated Th17-Polarizing Cytokine Production by BMDCs *In Vitro*

As signals transmitted from the luminal bacteria to the immune system are mainly through detection of bacteria by dendritic cells [35], BMDCs were chosen for our *in vitro* experiments. We examined whether *Prevotella* would stimulate BMDCs to produce Th17-polarizing cytokines. To this end, mouse BMDCs were obtained and stimulated with heat-killed *P. melaninogenica* and *P. copri* along with the TLR2 and TLR4 inhibitors to determine the effects of TLRs. LPS was used as the positive control, and PBS as the negative control. Phenotypes of BMDC were identified by flow cytometry analysis (Figure S2).

**Figure 6:**
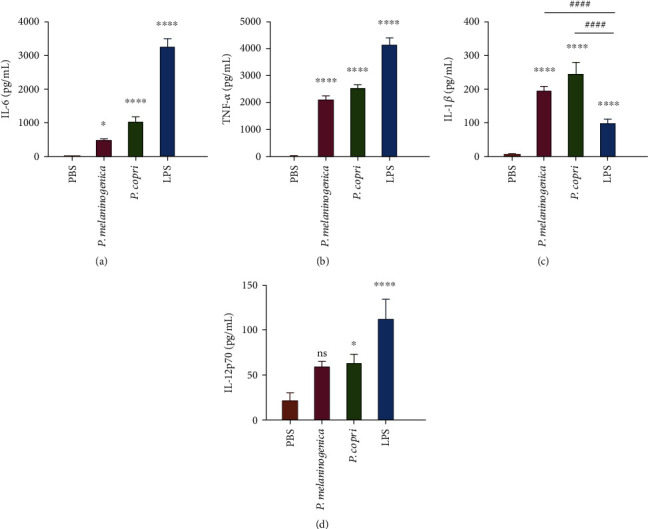
*Prevotella* induced Th17-polarizing cytokine production by BMDCs. (a–d) BMDCs were stimulated with heat-killed *P. melaninogenica* or *P. copri*. Then, cytokines in the cell culture supernatant were measured, including IL-6, TNF-*α*, IL-1*β*, and IL-12p70. One-way ANOVA was used to calculate *p* values, ∗ indicates statistical significance compared with the PBS control, and # indicates statistical significance compared with LPS. ^∗^*p* < 0.05, ^∗∗^*p* < 0.01, ^∗∗∗^*p* < 0.001, ^∗∗∗∗^*p* < 0.0001, and ^####^*p* < 0.0001.

Comparison of the production of various cytokines induced by the two species of *Prevotella* revealed that both of them were capable of inducing IL-6, TNF-*α*, and IL-1*β* (Figures 6(a)–6(c)). Notably, IL-6 and TNF-*α* were induced more dramatically by LPS than by *Prevotella*, whereas the opposite was true for IL-1*β* (Figures 6(a)–6(c)). For the induction of IL-12, there was no significant difference between the two species of *Prevotella*, although compared with PBS, *P. copri* showed statistical difference while *P. melaninogenica* did not (Figure 6(d)). These findings suggest that *Prevotella* can induce BMDCs to produce innate cytokines (i.e., TNF-*α* and IL-1*β*) and Th17- (i.e., IL-6 and IL-1*β*) and Th1- (i.e., IL-12) related cytokines. Additionally, IL-1*β*, which is indispensable for Th17 differentiation, can be induced more dramatically by *P. melaninogenica* and *P. copri* than by LPS.

**Figure 7:**
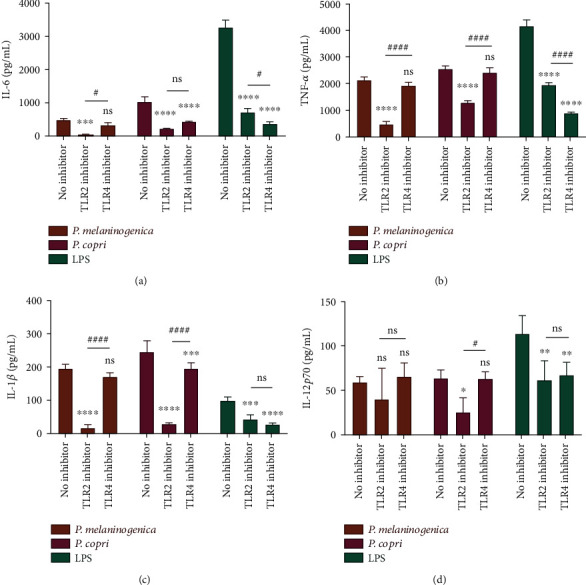
*Prevotella* activates TLR2-mediated Th17-polarizing cytokine production by BMDCs. (a–d) Inhibitors of TLR2 and TLR4 were used to investigate the involvement of Toll-like receptors in cytokine production by BMDCs induced by *Prevotella*. Data are presented as the mean ± SD. The *p* values were calculated using two-way ANOVA, ∗ indicates statistical significance between the TLR2 or TLR4 inhibitor-treated cells and the no-inhibitor control, and # indicates statistical significance between the TLR2 and TLR4 inhibitors. ^∗^*p* < 0.05, ^∗∗^*p* < 0.01, ^∗∗∗^*p* < 0.001, ^∗∗∗∗^*p* < 0.0001, ^#^*p* < 0.05, and ^####^*p* < 0.0001.

To determine the mechanisms involved, we investigated the roles of TLRs in the observed effects of *Prevotella* strains on BMDCs. C29 and TAK-242 were used as inhibitors of TLR2 [31] and TLR4 [32], respectively. For *P. melaninogenica* stimulation, IL-6, TNF-*α*, and IL-1*β* production by BMDCs was TLR2-dependent. This was also the case for *P. copri*, with the exception of *P. copri* inducing BMDCs to produce IL-6, where cytokine production was mainly dependent on TLR2 but with some contribution by TLR4 (Figures 7(a)–7(c)). Moreover, the induction of IL-12 by *P. copri* was completely mediated by TLR2 (Figure 7(d)). These data indicate the involvement of TLR2 on BMDCs in driving the differentiation of the Th17 subset upon recognition of *Prevotella*.

## 4. Discussion

Th17 cells are rich in the intestines in a stable state but are not found in the intestines of germ-free mouse, suggesting that this subgroup is produced through the reaction to the intestinal flora [36]. While numerous studies have documented associations between *Prevotella* and Th17 cells in many inflammatory diseases [17, 24], the underlying interaction between *Prevotella* and intestinal Th17 cells at a steady state was not previously known. The current study has identified two species of *Prevotella* that can induce robust Th17 cells in the murine colon, providing potential targets for the treatment of diseases related to Th17 responses.

Generally, two classic mouse models are used to detect the role of the intestinal flora in immunity. Germ-free mice are born and raised in aseptic conditions, resulting in abnormal immune functions due to the deprivation of the microbiota involved in the immune system education [33]. But for the antibiotic-treated mice, the situation is quite different. With the transiently depleted microbiota, they possess a mature immune system [34], So we used the ABX-treated mice for further study (Figure 1(a)). A previous study demonstrated that treatment with antibiotics significantly reduced intestinal Th17 cells in newborn mice [8]. In accordance with previous findings [37, 38], our data suggest that ABX-treated mice have a paucity of Th17 cells in both the colon and MLN (Figure 1).

After inoculation, we validated the abundance of *Prevotella* in the intestinal microbiota of mice by 16s rDNA sequencing (Figure 4). The composition of the intestinal bacterial communities demonstrate that, indeed, the major bacterial phyla are represented analogously between the *Prevotella*-gavaged mice (WT+PM and WT+PC) and the control mice (WT+BM) (Figure 4, Figure S1). These data indicate that the induction of intestinal Th17 cells is not dominated by the existence of diverse bacteria, but rather by the existence of specific bacteria (i.e., *Prevotella* in the current study).

The IFA of colon sections as well as flow cytometry analysis of the MLNs confirmed the robust production and accumulation of colonic Th17 cells induced by *P. melaninogenica* and *P. copri* (Figure 3). Thus, we conclude that *Prevotella* is among the symbiotic gut microbiota, specifically inducing the production of intestinal Th17 cells. *Prevotella* is especially common in non-Westerners who consume a plant-rich diet [21]. As one of the three gut enterotypes [39], the abundance of *Prevotella* in the gut changes with age [40, 41]. A study on metagenomic sequencing of intestinal microbes from 281 children at 6-9 years of age identified the enterotype driven by a high abundance of the genus *Prevotella* (*n* = 74), and *P. copri* is the dominant contributor [39]. Thus, *P. copri* and *P. melaninogenica* are likely to be common Th17-inducing bacteria in humans.

IL-17A is an important effector cytokine derived from Th17 cells that has a direct effect on epithelial cells, inducing antimicrobial peptides and recruiting neutrophils [42]. This is consistent with our observation that *Prevotella*-induced IL-17A is mainly located on intestinal epithelial cells (Figure 3(b)).

Th17 cells are believed to have dual roles in human health, so are microbiota-dependent Th17 cells. On the one hand, they appear to support host mucosal defence through various mechanisms, including enhancing the integrity of the barrier, providing cross-protections against pathogens in the early stages of infection, and shaping the gut microbiota [43]. On the other hand, symbiont-driven intestinal Th17 responses have been linked to IBD and some extraintestinal autoimmune diseases [20]. Our findings from the detection of serum cytokines in *Prevotella*-colonized mice indicate that *Prevotella* does not influence the cytokine profile in serum except a part of Th17-related cytokines (Figure 5). We saw no increase in either Th1- (IFN-*γ* and IL-2) or Th2-related (IL-4) cytokines (Figure 5). Notably, despite similar Th17 induction, *P. melaninogenica* and *P. copri* exhibited differential effects on the serum cytokine profile. In contrast to *P. copri*, *P. melaninogenica* appeared to be an inducer of IL-10 *in vivo* (Figure 5). Therefore, *P. copri* seems to be a purer Th17 inducer. Future studies are warranted to clarify whether *P. melaninogenica* induces the increase in IL-10 directly or through the modulation of community composition of the intestinal microbiota.

In addition, pathways activated by *Prevotella* have been elucidated preliminarily. It has been demonstrated that in response to bacterial contact, DCs are activated to secrete cytokines driving the unique differentiation and expansion of the CD4^+^ T cell in the intestine [44, 45]. Such *in vivo* conditioning of DCs by bacteria appears to extend outside the intestine such as BMDCs [46]. And our results revealed that both species of *Prevotella* (*P. copri* and *P. melaninogenica*) induced the production of IL-6, TNF-*α* and IL-1*β* by BMDCs (Figure 6). Among them, IL-6 and IL-1*β* have been reported to play crucial roles in the development of Th17 cells [47–49]. The Th17 differentiation process has previously been classified into two stages: a priming stage and a maturation stage [50]. IL-6 participates in the priming stage [51]. IL-1*β* has been verified to promote the expression of ROR*γ*t through the IRF4 pathway, resulting in IL-1-dependent Th17 cell polarization [52]. Moreover, another study showed that blocking of TNF-*α* markedly reduced the levels of IL-6, IL-1*β*, and IL-17 in patients with rheumatoid arthritis, indicating that Th17 cell differentiation is promoted by TNF-*α* through IL-6 and IL-1*β* [53]. All these findings suggest that *Prevotella* can induce Th17-driving cytokines by stimulation of BMDCs *in vitro*.

We further revealed that *Prevotella* mainly signals through TLR2 on BMDCs to induce the production of Th17-polarizing cytokines (Figure 7), which is in line with the previous report describing TLR2 as the main receptor involved in IL-1-driven Th17 responses through stimulation on antigen-presenting cells [15]. It is generally accepted that TLR4 mediates LPS signalling and that TLR2 mediates signalling of other cell surface components of bacteria, such as lipoprotein, peptidoglycan, and lipoarabinomannan [54]. However, it is reported that the LPS specimens prepared from *Prevotella* have chemical and biological characteristics that are different from those of LPSs from the Enterobacteriaceae [55]. A previous study has indicated that a *Prevotella* glycoprotein (PGP) extracted from *P. intermedia* activates monocytes in a TLR2-dependent way [56] and this observation is in support of our results.

**Figure 8:**
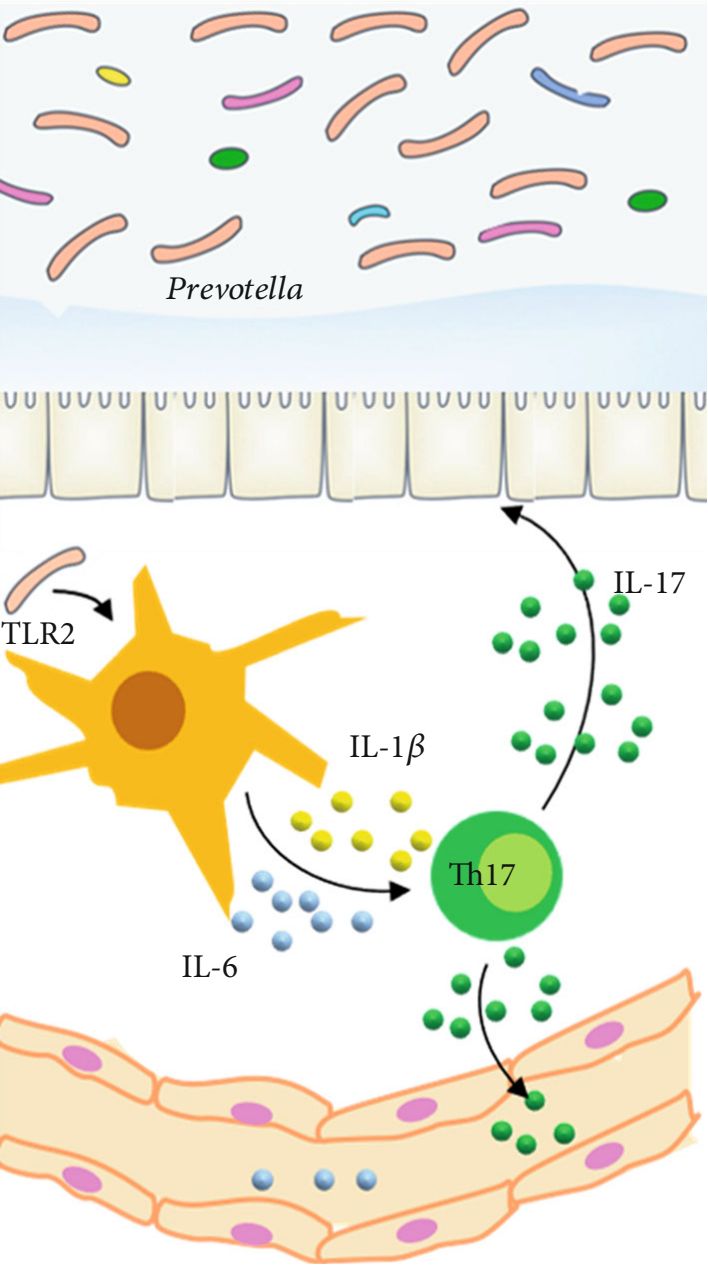
Mechanistic figure of the current study.

This study is exploratory, and more work remains to be done to better understand the mechanisms used by *Prevotella* and the effects caused by the accumulation of *Prevotella*-induced colonic Th17 cells. The current study provides the first demonstration that *P. copri* and *P. melaninogenica* can induce robust Th17 populations in the colon and the Th17-polarizing cytokines produced by DCs are mainly mediated by TLR2 (Figure 8). Given the ubiquitous distribution of *Prevotella* in the intestinal microbiota, it is highly plausible that therapeutic modulation of the *Prevotella*-regulated pathway will inform new attempts for enhancing mucosal immunity and reducing risk of inflammatory disease in susceptible hosts.

## Data Availability

Data supporting the findings of this study are available from the corresponding author (Prof. Yueyun Ma) on request.

## Supplementary Materials

Supplementary Materials Supplementary Table 1: primers for real-time PCR. Supplementary Figure 1: dynamic changes of relative abundances of Actinobacteria, Bacteroidetes, Firmicutes, and Proteobacteria in the gut microbiota of the three groups of mice. Supplementary Figure 2: phenotype of BMDCs analyzed by flow cytometry.
