# Supplementary material for: Prevotella Induces the Production of Th17 Cells in the Colon of Mice
Source: J Immunol Res. 2020 Nov 1;2020:9607328. doi: 10.1155/2020/9607328 (PMC7657696; doi:10.1155/2020/9607328)
Supplement: Supplementary Materials — Supplementary Table 1: primers for real-time PCR. Supplementary Figure 1: dynamic changes of relative abundances of Actinobacteria, Bacteroidetes, Firmicutes, and Proteobacteria in the gut microbiota of the three groups of mice. Supplementary Figure 2: phenotype of BMDCs analyzed by flow cytometry. [file 9607328.f1.pdf]

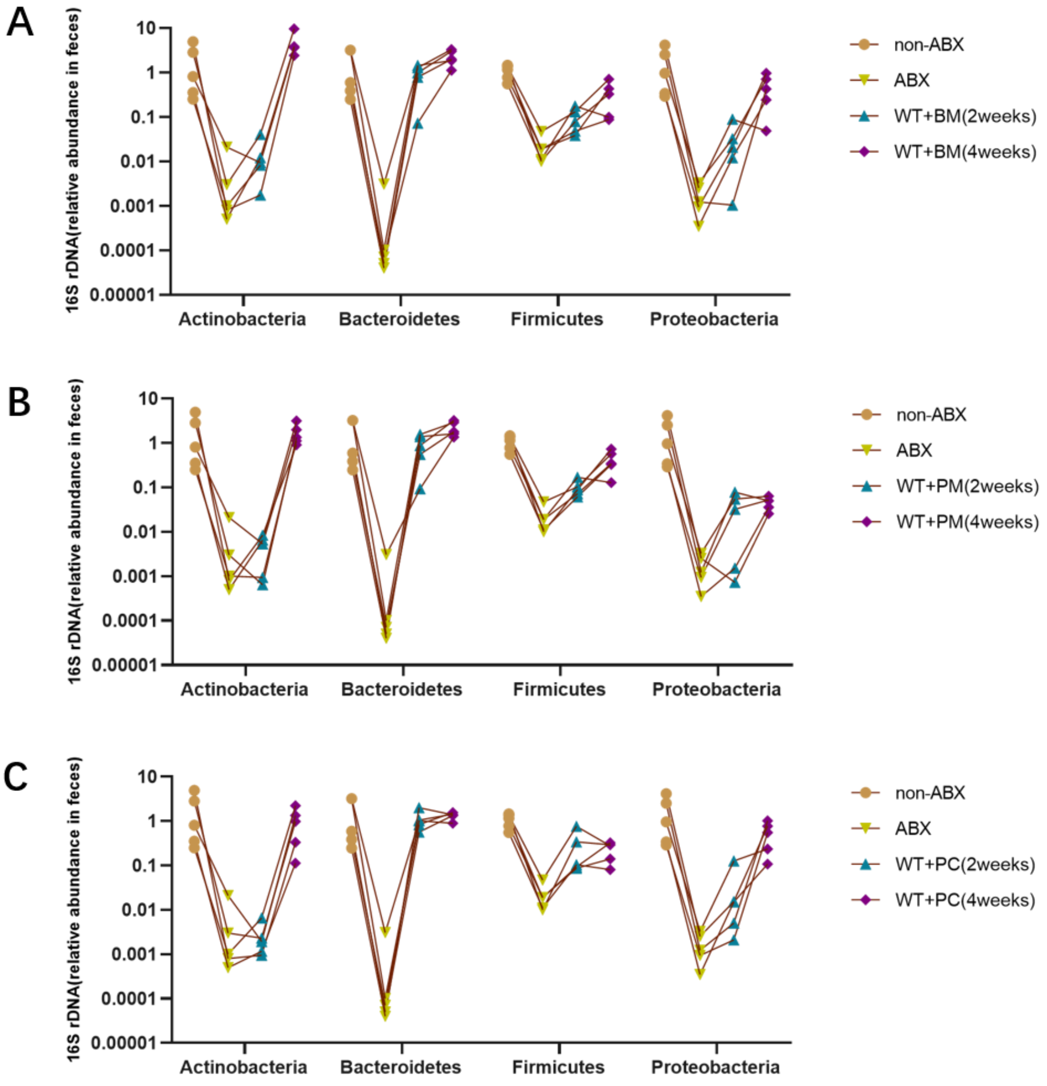

Supplementary Fig. 1. Dynamic changes of relative abundances of Actinobacteria, Bacteroidetes, Firmicutes and Proteobacteria in gut microbiota of three groups of mice.

Relative abundances of four phyla (Actinobacteria, Bacteroidetes, Firmicutes and Proteobacteria) in fecal microbiota was detected at four different time points (before ABX, after ABX, inoculation for 2 weeks and 4 weeks) by qPCR in mice gavaged with blank medium (A), *P. melaninogenica* (B) and *P. copri* (C).

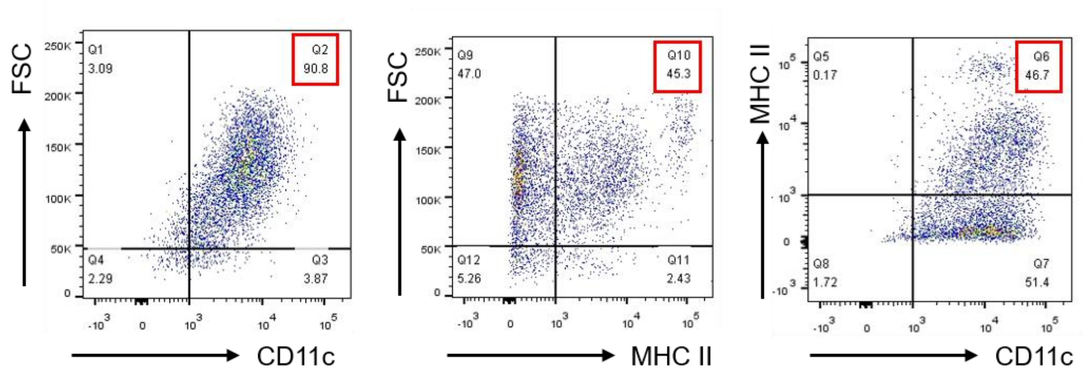

Supplementary Fig. 2. Phenotype (MHC II and CD11c) of BMDCs analyzed by flow cytometry.

After being purified by positive selection using anti-CD11c-microbeads, BMDCs were stained with Anti-mouse MHC Class II-APC and CD11c-PE before tested by flow cytometry. **(A)** $CD11c^+$ BMDCs **(B)** MHC II^+ BMDCs **(C)** $CD11c^+$ MHC II^+ BMDCs.

Supplementary Table1

Table 1: Primers for Real-time PCR.

Gene	Forward (5´--3´)	Product(bp)	Tm (℃）
gapdh	GGTGAAGGTCGGTGTGAACG<br>CTCGCTCCTGGAAGATGGTG	233	56
il-17a	TGAAGAGGGAGCCTGAGAGC<br>AGTAAGTTTGCTGAGAAACGTGGG	159	60
Universal	AAACTCAAAKGAATTGACGG<br>CTCACRRCACGAGCTGAC	136	60
Actinobacteria	TGTAGCGGTGGAATGCGC<br>AATTAAGCCACATGCTCCGCT	277	60
Bacteroidetes	GTTTAATTCGATGATACGCGAG<br>TTAASCCGACACCTCACGG	122	60
Firmicutes	GGAGYATGTGGTTTAATTCGAAGCA<br>AGCTGACGACAACCATGCAC	126	60
Delta- and Gammaproteobacteria	GCTAACGCATTAAGTRYCCCG<br>GCCATGCRGCACCTGTCT	189	60
